# Identification of novel candidate genes for follicle selection in the broiler breeder ovary

**DOI:** 10.1186/1471-2164-13-494

**Published:** 2012-09-19

**Authors:** Neil A McDerment, Peter W Wilson, David Waddington, Ian C Dunn, Paul M Hocking

**Affiliations:** 1 The Roslin Institute & R(D)SVS, University of Edinburgh, Easter Bush, Midlothian, EH25 9RG, Scotland, UK

**Keywords:** Broiler breeder, Multiple ovulation, Follicle development, Ovary, Microarray

## Abstract

**Background:**

Broiler breeders fed *ad libitum* are characterised by multiple ovulation, which leads to poor shell quality and egg production. Multiple ovulation is controlled by food restriction in commercial flocks. However, the level of food restriction raises welfare concerns, including that of severe hunger. Reducing the rate of multiple ovulation by genetic selection would facilitate progress towards developing a growth profile for optimum animal welfare.

**Results:**

The study utilised 3 models of ovarian follicle development; laying hens fed *ad libitum* (experiment 2) and broiler breeders fed *ad libitum* or a restricted diet (experiments 1 & 3). This allowed us to investigate gene candidates for follicular development by comparing normal, abnormal and “controlled” follicle hierarchies at different stages of development. Several candidate genes for multiple ovulation were identified by combining microarray analysis of restricted vs. *ad libitum* feeding, literature searches and QPCR expression profiling throughout follicle development. Three candidate genes were confirmed by QPCR as showing significant differential expression between restricted and *ad libitum* feeding: FSHR, GDF9 and PDGFRL. PDGFRL, a candidate for steroidogenesis, showed significantly up-regulated expression in 6–8 mm follicles of *ad libitum* fed broiler breeders (*P* = 0.016), the period at which follicle recruitment occurs.

**Conclusions:**

Gene candidates have been identified and evidence provided to support a possible role in regulation of ovarian function and follicle number. Further characterisation of these genes will be required to assess their potential for inclusion into breeding programmes to improve the regulation of follicle selection and reduce the need for feed restriction.

## Background

Sexually mature hens are capable of ovulating almost once per day for the duration of their period of lay. However, although modern breeding programmes consider many more traits [1], in early breeding programmes, commercial broiler breeders that were selected primarily for rapid growth and high meat yield produced fewer eggs [2,3]. The result of that selection pressure is that when fed *ad libitum*, broiler breeders produce multiple ovulations due to the development of a numerically large ovarian hierarchy [4]. While many ova from multiple ovulations are lost into the body cavity, some result in the production of double- or multiple-yolked eggs, or eggs with defective shells [3]. Additionally *ad-libitum* fed broiler breeders will become very heavy and develop various weight-related problems. Consequently, although juvenile broiler breeders are fed *ad libitum*, they are required to be limited to at least 40% of their natural nutritional intake as they reach sexual maturity to alleviate these problems [5]. However, this leaves the birds in a permanently hungry state. Feed restriction does however reduce follicle numbers in the ovarian hierarchy to a normal level [5], and consequently decreases the chance of multiple ovulation. Despite the benefits to the health of the birds, the degree of hunger experienced by the birds has raised concerns in some quarters as to the resulting impact on the birds’ welfare [3,6,7].

The mechanism by which feed restriction regulates follicle number has not been completely elucidated, though it is known to influence Gonadotrophin Releasing Hormone (GnRH) secretion within the hypothalamo-pituitary-gonadal (HPG) axis [8,9]. It is likely that food restriction exploits a natural mechanism. In wild birds, clutch size is affected by availability of food and the energy required to obtain it [10]. It is clear that food intake and related growth can influence follicular selection in the ovary of birds and the result of selection for these traits has affected the regulatory mechanisms that govern follicle selection within the ovary [11,12]. Modifying existing breeding programmes to select against multiple ovulation in commercial flocks is a potential route for reducing the requirement for feed restriction of broiler breeders.

Despite obvious differences in post-ovulatory mechanisms and specific hormone role reversals, the chicken ovulatory cycle is generally comparable with that of mammalian species [13-15]. The follicle is comprised of a series of membranes and highly vascularised cell layers that surround, support, and protect the oocyte [16]. Follicles within the avian ovary are ordered progressively by size from many follicles <1 mm in diameter to the pre-ovulatory or F1 follicle at around 40 mm [17]. Follicles can be grouped into 2 basic types, white follicles, which range from 1–7 mm in diameter, and yellow follicles, from approximately 8–40 mm, which are distinguishable by the deposition of yolk, that gives them their colour, and the increased vascularisation needed to support their growth [17]. Most follicles will not grow sufficiently to undergo ovulation and the majority <8 mm will become atretic. Normally under appropriate endocrine conditions following ovulation a follicle from the 6–8 mm pool is recruited into the pre-ovulatory hierarchy [18,19]. Once recruited and unless gonadotrophin support is removed these follicles are highly likely to mature and ovulate [17]. In pre-hierarchical development, Follicle Stimulating Hormone Receptor (FSHR) is the predominant gonadotrophin receptor [20]. However, once follicles are drafted into the hierarchy, this predominance shifts towards the Luteinising Hormone Receptor (LHR) [20].

Atresia in mammals predominantly occurs immediately prior to follicle recruitment [14,15,21,22]. Studies have shown that this occurs even in the absence of the pituitary-suggesting that regulation of pre-recruitment development is intra-ovarian once it has been initiated [14,15]. Pre-hierarchical development would therefore constitute the ‘normal’ lifespan of follicles, with progression to the hierarchy and subsequent ovulation being triggered externally [14,15,23].

External regulation of the avian ovary is managed through the HPG axis, with input signals being generated in the hypothalamus and relayed to the pituitary where hormone secretion is initiated [24]. The hypothalamus co-ordinates reproductive development and activity in response both to physiological and environmental signals, such as ovarian steroids [24] and photoperiod [25]. GnRH is released in pulses into the pituitary and the frequency and strength of these pulses modulate reproductive development [24] by inducing the production and release of LH and FSH from the pituitary [17,20,26-30]. While gonadotrophin signalling leads to cell growth and proliferation, as well as intrafollicular steroidogenesis and ovulation [13,17,29,31-33], mapping of the downstream pathways is less complete. Due to difficulties in isolating FSH from LH, avian FSH has proved challenging to study, and is not as well understood as the mechanisms involved in LH regulation [24]. Work has been carried out to investigate the roles of Bone Morphogenetic Proteins (BMPs) and Transforming Growth Factor B (TGFB) superfamily members in intrafollicular signalling, both in early and post-recruitment development [17] but less so in the intervening stages, notably the period of follicle selection and recruitment to the hierarchy.

The aim of this study was to investigate intraovarian regulation of follicle number in chickens by identifying gene candidates which have a potential role in either intrafollicular signalling or feedback mechanisms that affect the HPG axis. Ultimately this information will increase our understanding of the mechanisms by which dysfunction in broiler breeder hen ovaries occurs and may lead to genetic or alternative strategies to reduce dependence on food restriction.

## Methods

### Strategy

The research utilised comparisons of ovarian function between 3 sets of animals; 1) a broiler breeder line that was feed restricted (FR) or 2) fed *ad libitum* (AL) and 3) a line of layer hens fed *ad libitum*. The feed restricted broiler breeder and *ad libitum* layer share a comparable ovarian hierarchy. The 3 groups were used to examine changes in gene expression between key stages in follicle development in 3 experiments;

Experiment 1, gene expression in FR vs. AL broiler breeders was compared using microarray analysis of key stages of follicle development to determine the differences between birds with a low rate of follicle selection and birds with a high rate of follicle selection. Subsequent analysis of gene expression between these stages was carried out to identify changes as follicles progressed towards and through recruitment to the hierarchy. Two analytical approaches, in R [34] and BioLayout Express [35], were used to identify significant differences within these two comparisons.

Experiment 2, laying hens, having normal follicle hierarchies, were used to screen candidate genes from experiment 1 for changes in expression in a more detailed set of follicular stages. It was reasoned that genes showing large changes around the stage associated with follicle selection would be the most likely to be involved in recruitment.

Experiment 2, laying hens, having normal follicle hierarchies, were used to screen candidate genes from experiment 1 for changes in expression in a more detailed set of follicular stages. It was reasoned that genes showing large changes around the stage associated with follicle selection would be the most likely to be involved in recruitment.

### Birds and sampling: broiler breeders

Female Ross 308 broiler breeder chicks (n = 16) were reared for experiment 1 following management manual guidelines [36] with photoperiod rising to 16 L:8D by 25 weeks of age. At 29 weeks of age half the birds were allowed *ad libitum* access to feed and all were killed 2 weeks later. Sample collection was staggered over 2 days and was carried out 11 to 16 h after dusk. Birds were selected from a larger population at post-mortem to represent extreme ovarian phenotypes as regards numbers of hierarchical follicles. All birds had eggs present in the oviduct at sampling. At post mortem bird weight and the numbers of follicles greater >8 mm and between 5–8 mm diameter were recorded (Table 1). Tissues taken for probing the microarray were the F1 follicle, 5–6 and 6–8 mm follicles and the ovarian anterior stroma. 5–6 and 6–8 mm follicles were chosen as it is at this stage that the key changes are believed to occur [18,37]. Whole follicles were taken with yolks removed from hierarchical follicles. Female Ross 308 broiler breeder chicks (n = 23, 12 AL, 11 FR) were raised and sampled under the same conditions as above for experiment 3, with the additional inclusion of the smallest hierarchical follicle amongst the tissues taken.

**Table 1:** Trait means in broiler breeders from experiment 1

**Variable**	** Restricted**		*** Ad-libitum***		**P-value**
	**Mean**	**SEM**	**Mean**	**SEM**	
**Body Weight (Kg)**	2.89	±0.11	3.79	±0.07	<0.001
**Number of Follicles >8 mm**	5.62	±0.18	10.25	±0.37	<0.001
**Number of Follicles 5–8 mm**	10.6	±1.5	12.2	±1.4	NS
**Ovarian Stroma Weight (g)**	4.55	±0.31	6.91	±0.38	<0.001

### Birds and sampling: layers

Mature *ad libitum* fed White Leghorn layers (n = 8) were kept on a 28 h photoperiodic cycle (14 L:14D) for 3 weeks to synchronise ovulatory cycles. Sample collection was staggered over 3 days to allow all birds to be sampled approximately 20 h after dusk. All birds had eggs present in the oviduct at sampling. Follicles of each sample category were recorded (Table 2). Sampled tissues were the anterior stroma, pre-hierarchical follicles of diameter 1–4 mm, 4–5 mm increasing in 1 mm increments to 8 mm and the F6-F1 hierarchical follicles. Whole follicles were taken with yolks removed from hierarchical follicles.

**Table 2:** Trait means in White Leghorn layers from experiment 2

**Variable**	**Mean**	**SEM**
**Body Weight (Kg)**	2.05	±0.10
**Number of Follicles >8 mm**	6.00	±0.38
**Number of Follicles 5–8 mm**	12.13	±2.22
**Ovarian Stroma Weight (g)**	5.68	±0.10

### ARRIVE guidelines

Experiments were conducted after local ethical review and subsequent approval by the UK Government Home Office (Project licences 60/2926 and 60/3964) in accordance with the Animal (Scientific Procedures) Act 1986.

### RNA purification

Total RNA was isolated using Ultraspec II RNA kit (AMS Bioscience, Abingdon, UK) and the quality checked using an Agilent bioanalyzer (Agilent Technologies UK, Stockport, UK) for the RNA required for the microarray analysis and on a nanodrop (Thermo Scientific, Wilmington, DE, USA) for the samples to be used for QPCR.

### Microarray setup (experiment 1)

Broiler breeder samples were hybridised in 8 randomised pairs (FR v AL) for each tissue in a dye-swap microarray design using Cy3 and Cy5 (GE Healthcare, Little Chalfont, UK) dye labelled RNA. The chicken oligo microarray used was produced by ARK genomics ( http://www.ark-genomics.org) and contained 17 K features. Labelling was performed using a Stratagene Fairplay kit (Agilent Technologies Ltd, Stockport, UK) and hybridised using an automated GeneTAC hybridisation station (Genomic Solutions (Digilab), Huntingdon, UK).

### Preparation of cDNA

For confirmatory real-time quantitative PCR (QPCR) 1 μg of total RNA was reverse transcribed using a First Strand Synthesis Kit (GE Healthcare, Little Chalfont, UK) for Experiment 2. Experiment 3 used the High Capacity cDNA Reverse Transcription Kit (Applied Biosystems (Life Technologies), Paisley, UK). Primers for all candidates were designed using the Primer3 program [38]. All primer pairs were designed to produce products of between 150 bp and 250 bp and to be intron-spanning. All primer pairs were tested using both standard FastStart and SybrGreen PCR reagents and conditions. Production of single products was confirmed by both gel electrophoresis and by examining the dissociation curve and PCR products were confirmed by sequencing.

### QPCR expression profiling (experiment 2)

Candidate genes were initially screened to determine which showed greatest variation in expression between stages of follicle development using two 4-bird pools of anterior stroma, 5–6 mm, 6–8 mm, and F4 material taken from layers. Greatest variation between consecutive tissues was estimated using the following formula (assuming an efficiency >80%).

(1) $$2.5\left(\frac{\left(40-Ct2\right)-\left(40-Ct1\right)}{10}\right)$$

All primer pairs were subsequently shown to operate at estimated efficiencies of between 94-112%. This was done to prioritise candidates for comprehensive profiling. All sampled tissues were used for comprehensive profiling: QPCR for each candidate was run across 2 plates (4 birds per plate) and each plate was replicated. Lamin B Receptor (LBr) values were used for normalisation. Primers are listed in Additional file 1: Table S1. QPCR was carried out on cDNA according to a Platinum Sybr green (Invitrogen) protocol with duplicates using a standard curve on an MX3000 Sequence Detection System (Stratagene). Controls (no template) were run for all primer pairs.

### QPCR validation of dietary effect (experiment 3)

Bird pairs (1 AL, 1 FR) were randomised over 4 plates. QPCR was carried out on cDNA as in Experiment 2 with a positive control sample run in triplicate across plates to normalise between plates. LBr values were used for normalisation. Primers are listed in Additional file 1: Table S1.

### Between-treatment statistical analysis (experiment 1)

All basic statistical analysis of microarray data was carried out in an R environment [34] using the Bioconductor Limma package [39] and the protocol outlined in [40]. The data was quantile-normalised and means were calculated for replicate spots. A split-plot ANOVA was used to estimate the between-treatment effect. A Mann-Whitney non-parametric *t*-test was used to validate the normalisation process. All microarray analysis was corrected for multiple testing using correction for False Discovery Rate (FDR) [41].

### Between-tissue statistical analysis (experiment 1)

For each probe, mean values were calculated for each bird-pair within the microarray to remove the dietary variable prior to between-tissue comparison. The datasets for the individual ovarian tissues were combined and then quantile-normalised within R prior to performing a Kruskal-Wallis one-way ANOVA to identify probes that showed significant differences between tissues. A threshold of *P* <0.01 was used.

### Between-tissue cluster analysis (experiment 1)

An expression file was created using normalised bird-pair mean intensity values from R. This consisted of annotation columns and 32 data columns representing the 4 ovarian tissues from the 8 bird pairs. BioLayout Express3D ( http://www.biolayout.org/) was used to analyse this data file. File construction and data analysis were carried out according to the protocol available from the website [35]. A Pearson correlation threshold of 0.9 was used in the initial analysis and the embedded clustering algorithm (MCL) was used to cluster genes by expression profile. Clusters were limited to n ≥ 3 where n = no. of probes.

### Candidate selection (experiment 1)

Candidates identified from the comparison of follicular tissues from the microarray were selected using a multi-level filtering system. This used, as the basis, genes that were shown to be significantly differentially expressed between 2 or more follicular stages in R, and also conformed to patterns of expression (expression profiles) within BioLayout that were considered as consistent with a role in follicle recruitment. In addition, probes identified within both the Between-Treatment Statistical Analysis and the Between-Tissue Cluster Analysis in BioLayout Express were examined for supporting literature. Genes within follicle number QTL regions on chromosome 13 and the short arm of chromosome 4 [unpublished results of a low power genome scan] were also examined and those showing altered expression between *ad libitum* and restricted feeding in BioLayout Express consistently across bird pairs in at least 1 tissue (change in intensity >2000 units), with relevant supporting literature, were also included. A co-expression analysis was also carried out in Biolayout Express to identify genes that clustered with FSH Receptor (FSHR).

### Between-tissue statistical analysis (experiment 2)

QPCR datasets for each candidate gene were log-transformed using natural logarithms. An Analysis of Variance was run in GenStat [42], using the model containing fixed effects for Tissue within Bird and Plate as a Blocking factor.

### Treatment by tissue statistical analysis (experiment 3)

Following correction for plate effect and normalisation, replicate means were calculated and log-transformed. A Linear Mixed Model (REML) was run in GenStat [42] using a model with effects for Tissue x Treatment with Bird as the Block effect.

## Results

### Experiment 1: microarray examination of effects of release from feed restriction

Release from feed restriction to *ad libitum* feeding for 2 weeks increased the body weight of hens, the number of yellow yolky follicles (>8 mm), and ovarian stroma weight (Table 1). Although many genes showed altered expression as a result of release from feed restriction in ovarian tissues, the level of statistical significance did not justify further investigation on this evidence alone.

### Experiment 1: between-tissue analysis

Statistical analysis of the data on a between-tissue basis, after removing the dietary effect, produced 5571 probes with *P*-value of <0.01 and an additional 1149 at <0.05, indicating a significant difference between one or more of the tissues.

**Figure 1:**
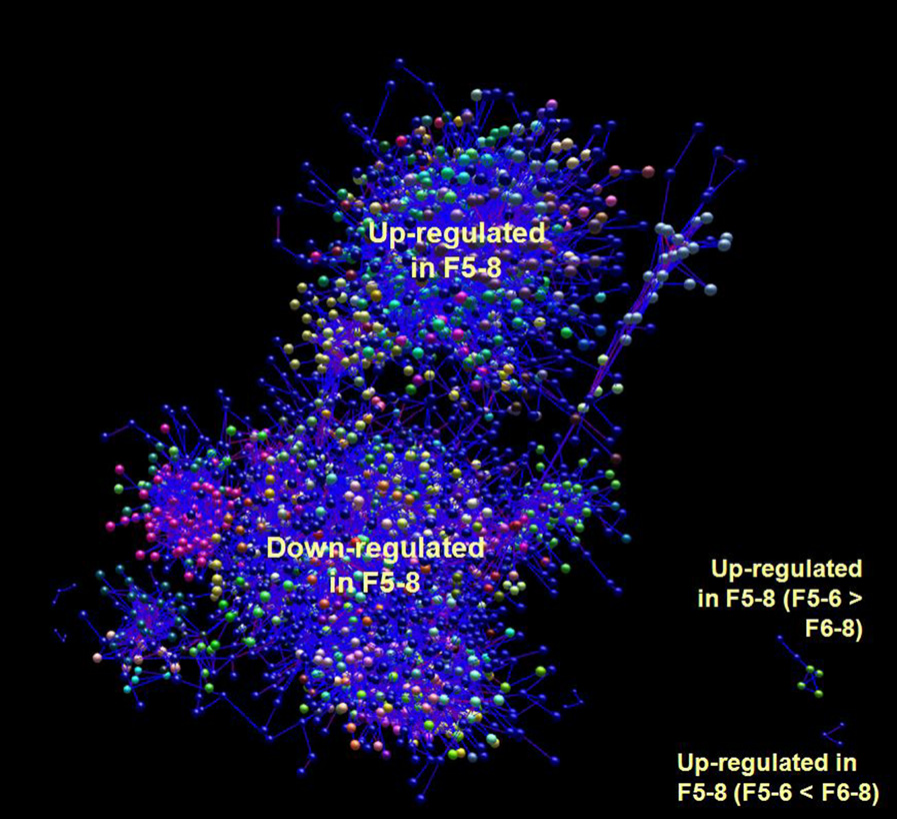
**3D Network of probes derived from the between-tissue expression file as visualised in BioLayout Express3D.** Nodes within the network represent probes from the array. Probes are clustered based on Pearson correlations calculated in BioLayout Express. General locations of selected expression profile types (as seen in Figure 2) are indicated.

**Figure 2:**
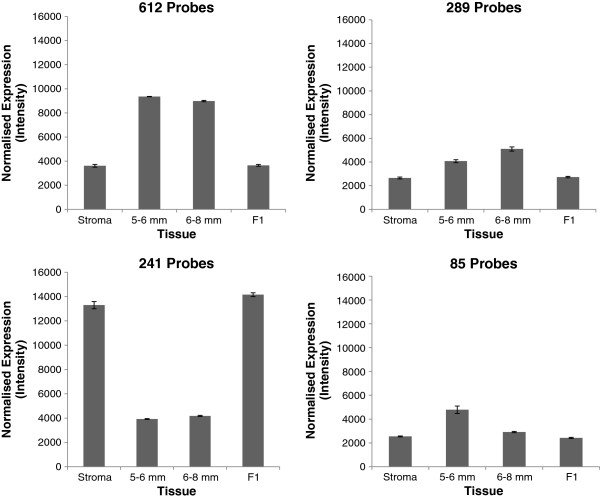
**The four cluster expression profiles from BioLayout Express relevant to follicle selection.** Individual cluster expression profiles illustrating the patterns of expression which show changes relevant to the critical time of follicle selection. Each plot represents the mean expression at the different stages of follicular development for all probes within a single cluster representing each profile type. Each profile type was exhibited by several clusters. Numbers of probes refer to total probes exhibiting each profile type within the dataset that were also significant according to the Kruskal-Wallis Test carried out in R.

An initial network of 5189 probes was produced from the BioLayout Express analysis (Figure 1). MCL produced 260 clusters of probes, 101 of which exhibited expression profiles indicative of a possible role in follicle development due to the changes in expression between tissue stages. Within the latter, 4 distinct profile types could be observed, as represented in Figure 2. No probe in the profile lists from BioLayout Express had a Kruskal-Wallis (K-W) *P* value of >0.002 which suggests that BioLayout Express is successfully filtering out the vast majority of non-significant data at point of entry. With this double-filtering by the Kruskal-Wallis *P*-value and the BioLayout Express Pearson correlation, the number of probes under consideration was reduced to 1,227. The Kruskal-Wallis/BioLayout-filtered probe lists were compared with the Top-50 probes (by *P* value) from the feed restriction vs. *ad libitum* feeding comparison within tissues in order to identify any genes common to both analyses. This process identified 13 candidate genes (Table 3). No apparently common related function for these genes could be identified through literature mining, although a number have functions of potential relevance to different aspects of follicular development. Genes located within the two QTL regions for follicle number were also identified and those with potentially differential expression between *ad libitum* and feed restricted diets, or that had a documented function of interest, were also included in the list of candidates (Table 4).

**Table 3:** Genes identified from comparison of the results from experiments 1 and 2

**FR vs. AL Microarray Gene List**	**5-6 mm Top 50**	**6-8 mm Top 50**	**F1 Top 50**	** Stroma Top 50**	
**BioLayout Profile Gene List**	**Up in 5–7 mm**	**Up in 5–7 mm**	**Up in 5–7 mm**	**Up in 5–7 mm**	**Down in 5–7 mm**
**Genes common to both lists**	MYO1C	MYO1C	GRP	RIGG01740	POSTN
	YAP1	GULP1	ZNF593	SPTY2D1	PDGFRL
		RIGG03908	MAMDC2		TBC1D13
		RIGG05331			

**Table 4:** Genes within follicle number QTL on chromosomes 4 and 13 where BioLayout analysis predicted up-regulation in response to ad libitum feeding

**Symbol**	**ARKClone ID**	**K-W P Value**	**Known Function (EntrezGene)**
ADRA1B	RIGG07717	0.002	Multicellular Organism Development & Cell Growth
CAMK2A	RIGG08243	NS	Ca2+ Signalling & Cell Cycle
FGF13	RIGG08380	0.003	Embryonic Development & Cell Growth
FOXI1	RIGG10898	NS	Multicellular Organism Development
GDF9	RIGG13716	<0.001	Folliculogenesis
PAK3	RIGG08561	0.012	Multicellular Organism Development
PPARGC1B	RIGG10412	NS	Oestrogen Receptor Binding
SLIT3	RIGG06586	NS	Extracellular pro-apoptotic signalling
VDAC1	RIGG13395	<0.001	Ca2+ Signalling & Regulation of Apoptosis

Subsequent co-expression analysis of the BioLayout Express dataset identified 1 gene of possible interest. Motile sperm domain containing 1 (MOSPD1) was clustered with FSHR in follicular tissues.

### Experiment 2: investigation of candidates by QPCR in layers

Table 2 shows trait means for the White Leghorns used in experiment 2 for expression profiling of candidate genes selected from experiment 1. It is clear from these results that broiler breeders under restricted feeding (from Table 1) have a similar follicular hierarchy to that of layers.

Of an initial 60 genes under consideration on the basis of their expression pattern in BioLayout Express, 37 were confirmed as being of potential interest, including 10 of the 13 differentially expressed genes identified by cross-referencing results from the comparison of tissues and feed regime. Added to this list were 22 literature-sourced genes, 3 of which (FSHR, SMAD3 and TGFBR1) were carried through to experiment 3.

**Figure 3:**
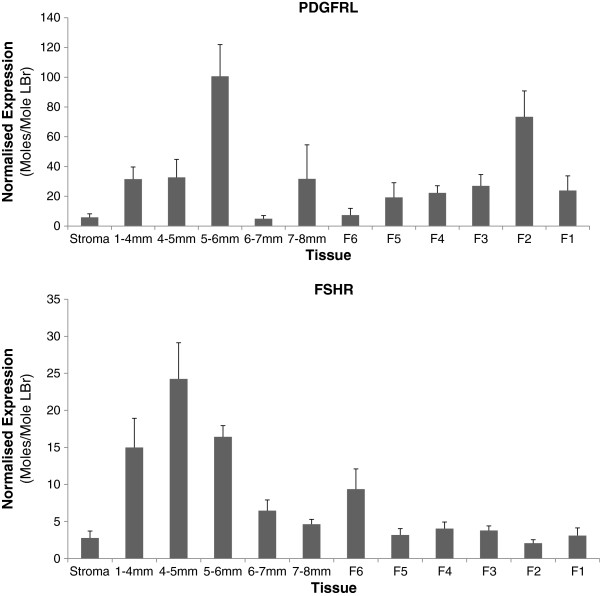
**QPCR expression profiles of primary candidate genes in layers (n = 8) from experiment 2.** Primary candidate status was determined at the conclusion of experiment 3. Values represent moles/mole of LBr. Note differences in the scale of the Y axis.

**Figure 4:**
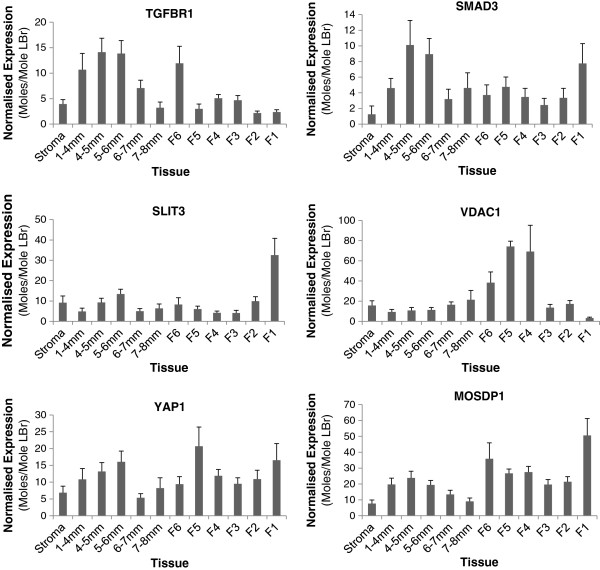
**QPCR expression profiles in layers (n = 8) for secondary candidate genes from experiment 2.** These genes were included for their documented function (Table 5) and because they demonstrated the greatest changes in expression during the initial screen in experiment 2. Values represent moles/mole of LBr. Note differences in the scale of the Y axis.

Genes with the greatest estimated fold change in the initial screen were ranked by the breadth of supporting evidence from the available literature, their BioLayout profile and experimental evidence. The 12 top ranking genes were selected as primary candidates for detailed QPCR analysis. Table 5 summarises this information while the QPCR-derived expression profiles for each assay are displayed in Figures 3 and 4, and Additional file 2: Figure S1. All 12 genes showed significant differential expression between tissues (*P* ≤ 0.017).

**Table 5:** Summary of supporting evidence for primary candidates examined in experiment 2

**Gene**	**QPCR P value**	**BioLayout Profile**	**QTL Chromosome**	**AL vs. FR (Predicted)**	**Literature**
**FSHR**	0.001				Key mediator of reproductive signalling
**TGFBR1**	<0.001				Key mediator of cell growth + survival
**SMAD3**	<0.001				Promotes Cell Survival
**SLIT3**	0.001		Gga13	Up	Promotes Apoptosis
**PDGFRL**	<0.001	Down 5–8 mm		Up	Homologs involved in steroidogenesis
**VDAC1**	<0.001	Up in 5–8 mm	Gga13	Up	Central to pro-apoptotic signalling
**YAP1**	<0.001	Up in 5–8 mm		Up	Possible pro-cell survival signalling
**MOSPD1**	<0.001	Clustered with FSHR			Up-regulated in ovarian cancer
**KRT75**	<0.001	Down 5–8 mm			Possible Lipid Transport
**SPTY2D1**	<0.001	Up in 5–8 mm		Down	Unknown
**RIGG1740**	0.017	Up in 5–8 mm		Down	Unknown
**GULP1**	<0.001	Up in 5–8 mm		Up	Phagocytosis of apoptotic cells

### Experiment 3: validation of effect of AL vs. FR on gene expression in broiler breeders

**Figure 5:**
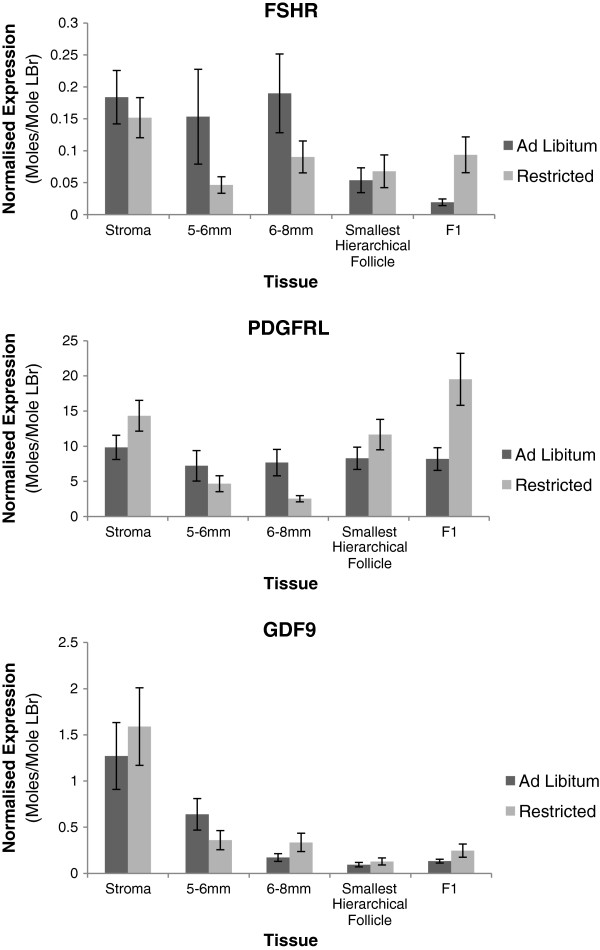
**QPCR expression profiles of primary candidates in broiler breeders (n = 23)from experiment 3.** These genes each showed significant differential expression between *ad libitum* and feed restricted broiler breeders. Details are summarised in Table 6.

Based on the results generated by experiments 1 and 2, several literature- and microarray-sourced candidates were selected for QPCR investigation of effect of *ad libitum* vs. restricted feeding. While all but two candidates showed significant between-tissue effects (*P* ≤0.027), none demonstrated a dietary effect that was significant across tissues. Three candidates, however, demonstrated significant dietary effects within specific tissues. Both literature-sourced candidates were down-regulated under *ad libitum* conditions, FSHR in the F1 Follicle (*P* = 0.018) and GDF9 in the Anterior Stroma and in 6-8 mm follicles (*P* = 0.005). PDGFRL, the novel candidate identified from the microarray was shown to be upregulated in response to *ad libitum* feeding in 6-8 mm follicles (*P* = 0.016). Expression profiles for these 3 genes can be seen in Figure 5. A summary of all significant effects can be found in Table 6.

**Table 6:** Summary of effects identified from investigation of dietary effect in broiler breeders (experiment 3)

**Gene**	**Candidate Source**	**Tissue*P *value**	**Treatment*P *value**	**Interaction*P *value**	**Interaction Effect**	**Tissue**
**GDF9**	Literature/QTL	<0.001	NS	0.005	Down in *Ad lib*	Stroma & 6–8 mm Follicles
**FSHR**	Literature	<0.001	NS	0.018	Down in *Ad lib*	F1 Follicle
**PDGFRL**	Microarray	<0.001	NS	0.016	Up in *Ad lib*	6-8 mm Follicles
**SLIT3**	QTL/Microarray	<0.001	NS	NS		
**SMAD3**	Literature	<0.001	NS	NS		
**BMPR2**	Literature	<0.001	NS	NS		
**TGFBR1**	Literature	0.005	NS	NS		
**YAP1**	Microarray	0.027	NS	NS		
**VDAC1**	QTL/Microarray	NS	NS	NS		
**MOSPD1**	Microarray	NS	NS	NS		

## Discussion

This research was based on the premise that, as broilers and layers have been genetically selected for different traits, the fact that adult broilers exhibit contrasting ovarian phenotypes under non-restricted conditions from layers is a result of genetic selection and there is ample evidence that this is the case [5]. Consequently, the effect of this genetic selection on expression of genes suspected of being important for reproductive development was undertaken, as regulation of their expression is likely to be the cause, directly or indirectly, of the phenotypic differences observed in broilers and layers. These are obvious targets for research to explain these differences but are also of fundamental interest in reproductive biology in a wider context. The study therefore used results from a range of experimental and analytical approaches to identify candidate genes for regulation of follicle selection and recruitment in the broiler breeder ovary. Two different approaches were used in complementary fashion to analyse the large dataset produced by the microarray. BioLayout Express, the more novel component of our analytical approach, has many built-in features that complement its use as a basic analytical tool. The most fundamental is the ability to visualise the data as a 3D network of datapoints that can be subsequently analysed by the internal clustering algorithm. The results can also be filtered by any available annotation or expression profile across samples to facilitate identification of possible associations between them. This makes it much easier to ask different questions of a single dataset and was an essential asset in switching focus between developmental stages and effect of nutrition. Using these different approaches to analyse the microarray data, as well as using information from different patterns of follicular development a number of genes that show changes in expression that would be consistent with a role in follicle selection have been identified. Three of these candidates have also been shown to exhibit altered expression levels as a result of *ad libitum* feeding.

From the 37 prioritised candidates considered for multi-level QPCR profiling, 10 candidates (including FSHR and TGFBR1 for validation of the approach) had sufficient experimental and/or literature-sourced evidence for basic hypothesis generation as to their role in follicle selection and recruitment. All of these candidates are associated with regulation of apoptosis [43-48], cell growth [20,49,50] and survival [51] or steroidogenesis [52] where there is any documented function. While these processes are all prerequisite to follicle survival, there is insufficient information at present to create a single model system incorporating all of our candidates, although FSHR, TGFBR1 and SMAD3, with the added possibility of MOSPD1, do interact with common signalling pathways. However, PDGFRL is by far the most intriguing novel candidate.

PDGFRL produces a product which is homologous with the functional domains of Platelet-derived Growth Factor Receptors that are involved in intrafollicular cell signalling associated with steroidogenesis in mice [52]. QPCR profiling in layers clearly shows significant (P <0.001) and substantial peaks in expression at the 5–6 mm and F2 stages, i.e. immediately prior to selection and ovulation respectively. This evidence would support a function in regulatory feedback mechanisms. The BioLayout Express profile from the broiler breeder microarray data suggests that the gene is downregulated in 5–7 mm follicles relative to the stroma and F1. This is consistent with the broiler breeder QPCR expression pattern for feed restricted birds. In contrast, the expression levels across tissues in *ad libitum* fed birds remain relatively constant. In *ad libitum* fed birds, where hierarchical follicle number is increased, the upregulation of PDGFRL expression, relative to feed restricted birds, suggests that it is likely to be in activation or upregulation of positive feedback signalling to the HPG axis. The observed downregulation in 5–7 mm follicles from broiler breeders is in marked contrast to the layers, which would support the hypothesis of potential dysregulation of part of the steroid-based feedback mechanisms, given what is already known of the PDGFR family in other species.

The QPCR results from experiment 2 show that FSHR and TGFBR1, genes known to be involved in follicle growth, peak in their expression during early prehierarchical development. This agrees with previous results [20,49]. Interestingly, both FSHR and TGFBR1 show a prominent peak at 8–10 mm, indicating that follicles immediately post-recruitment may have a heightened sensitivity to the ligands of these receptors at this stage. As both receptors activate pathways leading to cell growth, proliferation and differentiation, higher expression at those stages is not surprising. SMAD3, a known downstream signal mediator of TGFB family receptors demonstrates a very similar pattern of expression to TGFBR1. However, TGFBR1 and SMAD 3 did not show a significant effect in response to *ad libitum* feeding, whereas FSHR was significantly downregulated in the F1 follicle under *ad libitum* feeding. It is most likely that, as the lower FSHR expression shown in *ad libitum* fed broilers is more comparable with previous studies [53], the increased expression in feed restricted birds leads to negative feedback resulting from steroidogenic factors. Little functional significance has been placed on FSH control in the F1 and further investigation is warranted to explore the potential roles for FSHR in this follicle in light of the results reported here. MOSPD1 has been implicated in mesenchymal cell differentiation [50] and is upregulated in ovarian cancer [54]. BioLayout Express analysis showed it clustered with FSHR in broiler breeders and QPCR profiling in layers corroborated this. MOSPD1 is a membrane-associated protein [55] and may be involved in supporting or mediating signal transduction from the FSH receptor but further work will be required to determine this.

GDF9 was not identified through the original microarray analysis but was included due to its location near the putative QTL for follicle number in the chicken and because studies in sheep report an association between mutations in GDF9 and increased ovarian follicle number and ovulation rate [56,57]. Despite showing little change in expression between tissues in the initial screen in layers, GDF9 does show significant downregulation in response to *ad libitum* feeding in broiler breeders in the stroma and in 6–8 mm follicles (*P* = 0.005). In conjunction with results from mamalian studies this result would imply an inhibitory effect on follicle number. It is interesting to note that the expression profile for GDF9 in broiler breeders, regardless of diet, is comparable with other species [58-61], indicating a high level of inter-species conservation for this gene. Reported inter-species sequence conservation from the UCSC Chicken Genome Browser supports this, with sheep being most comparable in terms of exon coverage. Further investigation is underway to determine if there are mutations in the chicken, as there are in sheep, that might be associated with multiple ovulation.

SLIT3 and VDAC1 have both been shown to be involved in pro-apoptotic signalling [43-46] and are located in the putative QTL for follicle number on chromosome 13. SLIT3 is also involved in ovary and follicle development in sheep [62] and its expression profile in layers is consistent with phases of increased apoptosis. Expression profiling of SLIT3 and VDAC1 in BioLayout Express for broiler breeders is consistent with the layer QPCR profiling. However, there was no significant differential expression either between tissues or dietary regimes in the broiler breeder QPCR validation for either candidate.

YAP1 is believed to be involved in cell survival signalling through regulation of the p53 signalling pathway [51]. The BioLayout Express profile of the broiler breeders suggests that it is upregulated in late prehierarchical follicles. However, profiling in layers shows higher expression in early prehierarchical follicles and the F5 follicle, where pro-survival signals would be expected to occur more prominently. Upregulation in *ad libitum* fed birds could not be validated by QPCR. This does not negate a role for YAP1 in follicular development, however it is unlikely to be responsible for multiple ovulation in broiler breeders.

GULP1 is expressed in macrophages and is involved in engulfment of apoptotic cells [47,48]. Profiling in layers is consistent with this activity. While this is not likely to be a candidate for follicle selection, it does highlight the transitional stages of the follicle as it progresses through development.

RIGG1740, KRT75 and SPTY2D1 were also investigated for a potential role in follicle development, however their expression profiles in layers, in conjunction with the level of available evidence (summarised in Table 5) did not indicate a central role in follicle recruitment.

### Conclusions and further work

Our prime candidates, PDGFRL, GDF9 and FSHR, although only indirectly linked, all have strong cases for further investigation. GDF9 and FSHR are not novel candidates, indeed, FSHR was intially included in this study as a form of positive control and the identification of a previously unreported dietary effect on its expression was unexpected. PDGFRL however, is a novel candidate, and its implicated role in regulation of steriodogenesis, along with its response to *ad libitum* feeding makes it of primary importance.

Further investigation of PDGFRL, as well as FSHR and GDF9, is clearly warranted, and would yield many valuable insights. For example, work to determine localisation of expression of PDGFRL within the different cell types in the follicle wall is clearly needed to further substantiate its proposed role in steroid-based feedback. Successful examination of these candidates should bring us one step closer to solving the problem of multiple ovulation and allow for the prospect of relaxing feed restriction to improve the welfare of broiler breeder chickens.

## Competing interests

The authors declare that they have no competing interests with regard to this work.

## Authors’ contributions

NM carried out between-tissue statistical and BioLayout Express analysis of the microarray data and subsequent RNA processing and QPCR validation in experiments 2 & 3. PW, ID & PH carried out tissue collection for experiment 1 & 3, and PW carried out RNA processing microarray sample preparation and QPCR validation for experiment 1. DW carried out between treatment statistical analysis of Experiment 1. NM, PW & ID carried out tissue collection for Experiment 2. PH and ID co-supervised and obtained funding for the Defra study and CASE Studentship. All authors read and approved the final manuscript.

## Supplementary Material

Additional file 1 **Table S1.** List of primers used for qPCR indicating accession numbers and genome orientation for reference sequences used for primer design. Click here for file

Additional file 2 **Figure S1.** QPCR expression profiles in layers (n = 12) for remaining candidate genes from experiment 2. These genes showed less well defined expression profiles in layers and had insufficient supporting evidence to justify continued investigation. Click here for file
