# Supplementary figures and images for: Identification of novel candidate genes for follicle selection in the broiler breeder ovary
Source: BMC Genomics. 2012 Sep 19;13:494. doi: 10.1186/1471-2164-13-494 (PMC3511242; doi:10.1186/1471-2164-13-494)

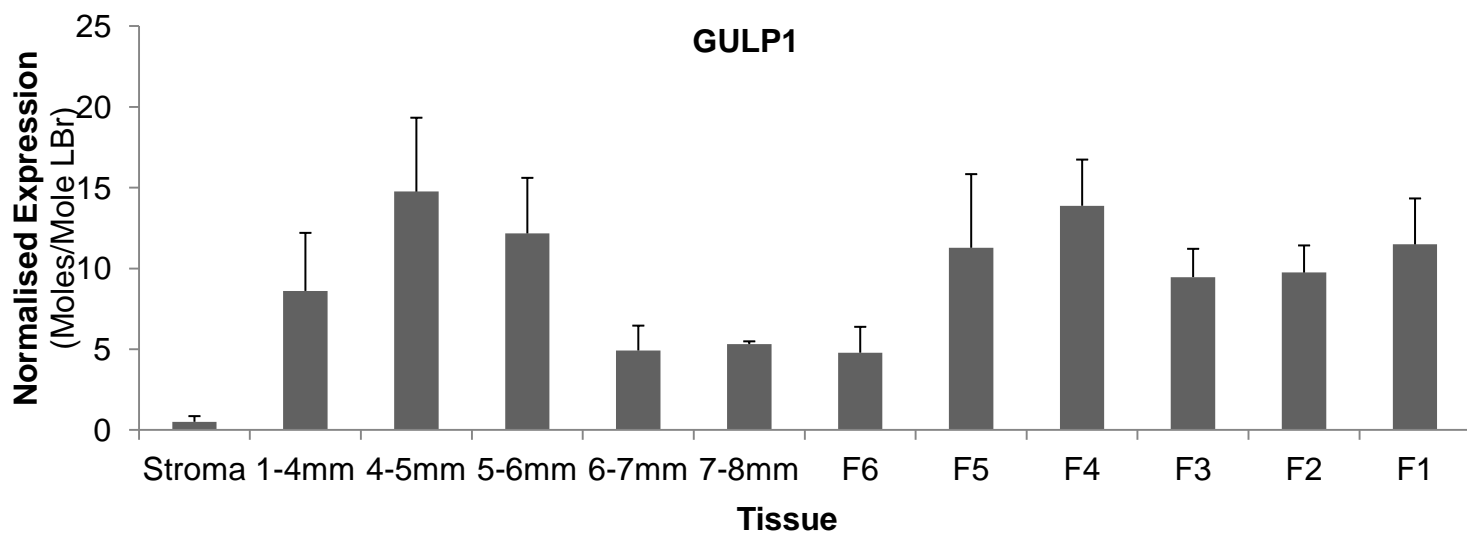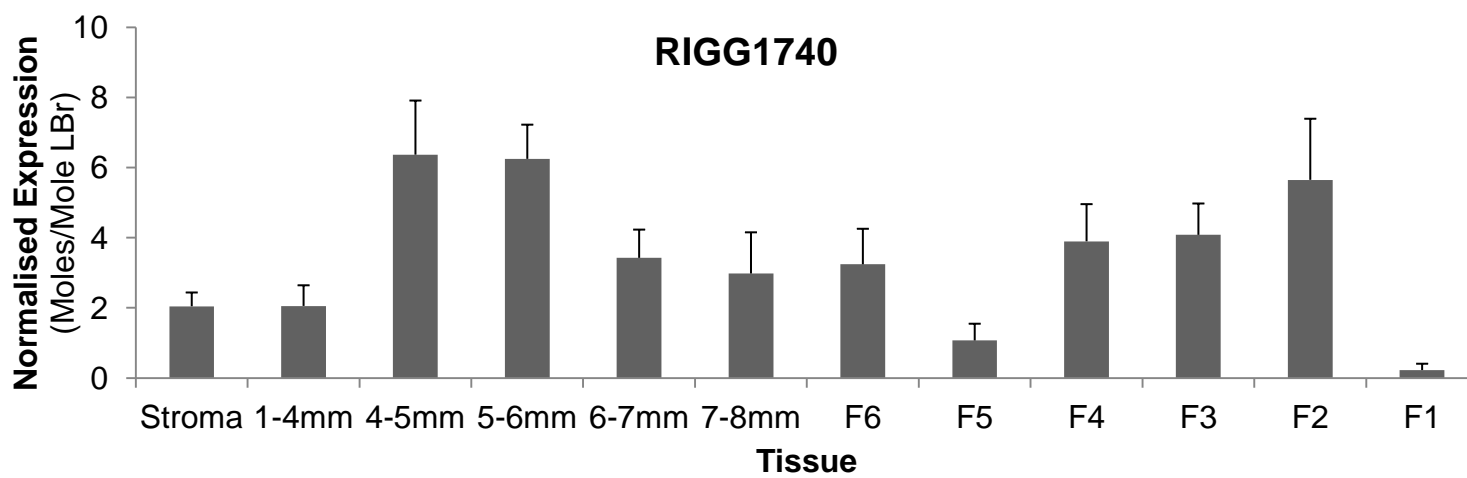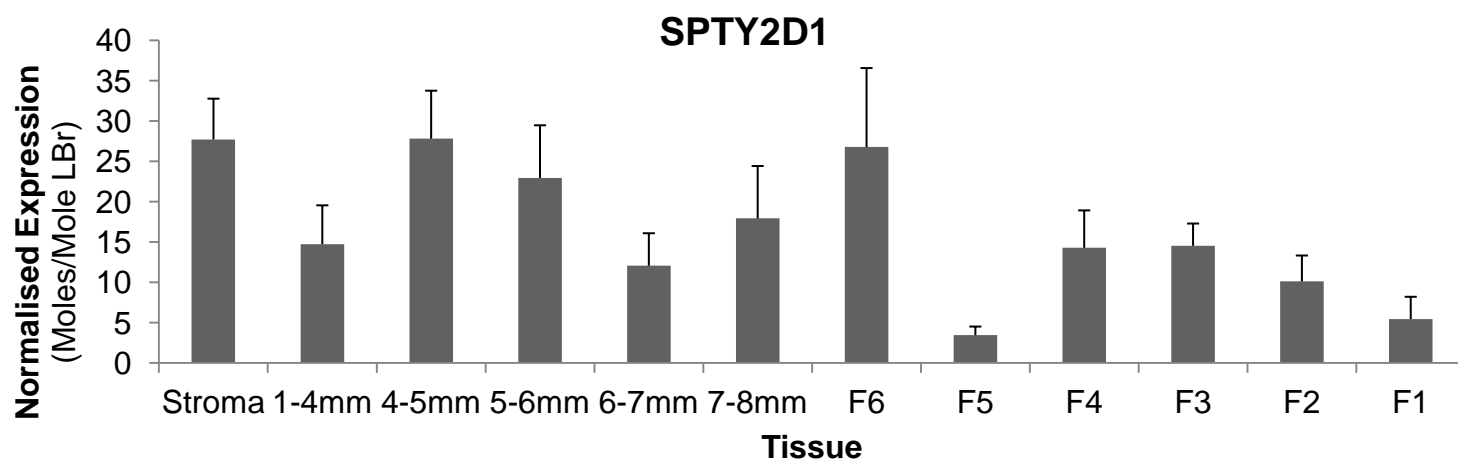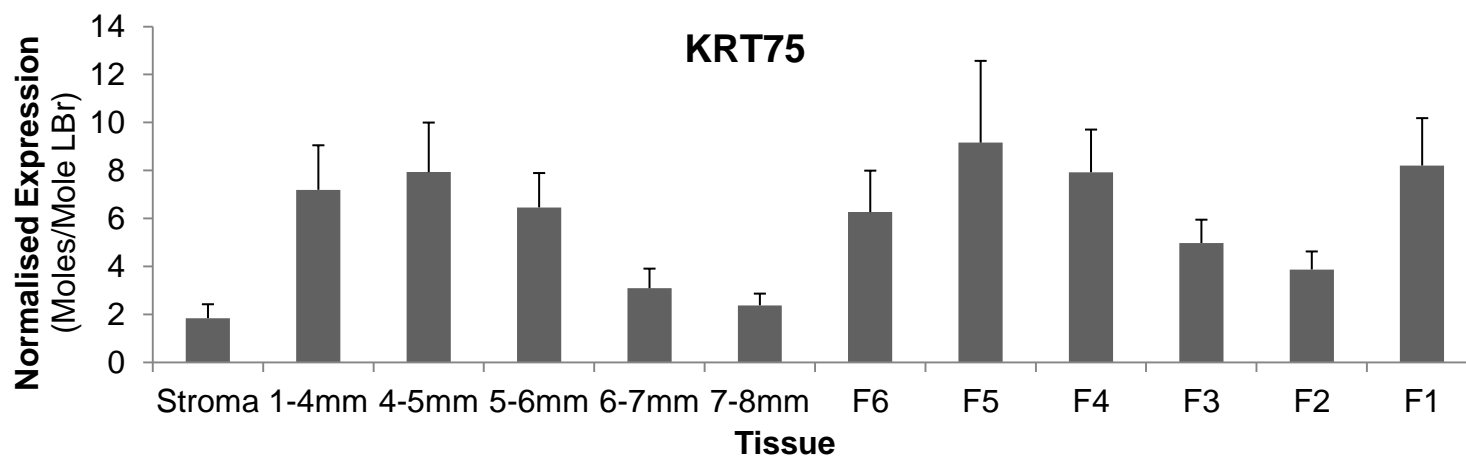

Supplement: Additional file 2 — Figure S1. QPCR expression profiles in layers (n = 12) for remaining candidate genes from experiment 2. These genes showed less well defined expression profiles in layers and had insufficient supporting evidence to justify continued investigation. [file 1471-2164-13-494-S2.pdf]
